# Supplementary material for: Prominin‐1 controls stem cell activation by orchestrating ciliary dynamics
Source: EMBO J. 2018 Dec 6;38(2):e99845. doi: 10.15252/embj.201899845 (PMC6331727; doi:10.15252/embj.201899845)
Supplement: Supplementary file 1 — Appendix [file EMBJ-38-e99845-s001.pdf]

Appendix

Appendix Figure Legends

Appendix Figure S1

Appendix Figure S2

Appendix Figure S3

Appendix Figure S4

Appendix Figure S5

Appendix Figure S6

Appendix Figure S7

Appendix Figure S8

Appendix Figure S9

Appendix Table S1

Appendix Figure S1. Ciliary dynamics in the mouse incisor stem cells and transit amplifying cells

A. Schematic illustration of a developing mouse incisor.

B. Representative IF staining of Bmi1 (red) on stem cell and transit amplifying cell regions in CLE. Sample is counterstained with DAPI (blue). Dotted lines: basement membrane. Yellow arrowheads mark approximate stem cell region boundaries. SCs, stem cells; TACs, transit amplifying cells;

C. Example of 3D reconstruction of a primary cilium based on the AcTub immunolabeling used for quantification. Original signal (left) and 3D reconstruction (right) of primary cilium (dotted line) are displayed.

D, E. Quantification and comparison of maximum cilia diameters (D) and density (E) in stem cell and transit amplifying cell regions on 4 samples for each. Statistics was performed using one-way ANOVA: **, $p < 0.01$. Data are represented as mean and standard deviation.

F. Representative immunofluorescent staining on Cre recombinase using specific antibodies, on the same batch of samples illustrated in Figs 1H and I.

G. Typical IF staining of AcTub (green) with Arl13b (red, lateral view) or Hdac6 (red, top view) along the stem cell-transit amplifying cell axis. Arrows indicate primary cilia.

Appendix Figure S2. Prom1 has a dynamic expression in the incisor CLE primary cilia and nuclei.

A. Representative IF staining of Prom1 using a specific antibody targeting its cytoplasmic C-terminal end (Biorbyt, Orb129549, green) on the stem cell and transit amplifying cell regions of a P7 lower incisor CLE. Sample is counterstained with

DAPI (blue). Dotted lines, basement membrane. SCs, stem cells; TACs, transit amplifying cells; Ams, ameloblasts.

B. Triple staining of primary cilia at stem cell to transit amplifying cell transition region using antibodies against AcTub (red) as a marker of primary cilium core, Arl13b (blue) and Prom1 (clone 13A4, green). The primary cilium is highlighted with dotted line. Note the asymmetric distribution of Prom1 along the primary cilium and its labeling outside primary cilium, which corresponds to microvilli.

C. 3D reconstruction of IF double staining of Prom1 (red) using a specific antibody targeting its cytoplasmic C-terminal end (Biorbyt, Orb129549) and E-Cadherin (ECad, green) of stem cell and transit amplifying cell regions in lower incisor CLE at P7. Samples are counterstained with DAPI (blue). Transparency of blue channel was set at 50% in left panels.

D. 3D reconstruction of IF staining of Prom1 (green) on transit amplifying cell region using a specific antibody targeting its extracellular loop (clone 13A4, green). Samples are counterstained with DAPI (blue). Transparency of blue channel was set at 0 or 70% as indicated.

E. Real-time RT-PCR profiling of Keratin 14 and Vimentin expression in the established CLESCs and CLE mesenchymal stem cells (CLEMSCs). The results are based on triplicated samples in arbitrary values after normalization for *GapDH*. Statistics was performed using one-way ANOVA followed by Bonferroni's test: **, $p < 0.01$. Data are represented as mean and standard deviation.

Appendix Figure S3. Prom1 regulates CLESCs maintenance and activation

A. Average cell density in the CLESCs region in the WT and *Prom1* KO mice. Three animals were analyzed per condition for each phenotype by quantifying DAPI

positive nuclei number at CLE region. Data are represented as mean and standard deviation.

B. Representative IF double staining of Sox2 (green) and Ki67 red) on the recombination samples illustrated in Fig 3F. Samples are counterstained with DAPI (blue). Dotted line indicates the CLE.

Appendix Figure S4. CLESCs renewal is compromised in the absence of Prom1

A. Plate images of passage 1 CLESCs extracted from WT vs. *Prom1* KO mice were cultured with or without SHH (100ng/ml) at density of 5,000 cells per well on 24 well plates. Cells were stained with crystal violet.

B. The knockdown efficiency of *Prom1* on the siProm1 treated CLESCs was determined by real-time RT-PCR analysis using specific primers (Appendix Table S1). The results are achieved on triplicated samples and are in arbitrary values after normalization for *GapDH*. Statistics was performed using one-way ANOVA followed by Bonferroni's test.: **, $p < 0.01$. Data are represented as mean and standard deviation.

C. Images of 24 plate's wells containing cells received siProm1 treatment. in combination with or without SHH (100ng/ml).

D, E. Average colony number (D) and size (E) per well on triplicated wells calculated from data (C). Data are represented as mean and standard deviation. Statistics was performed using one-way ANOVA followed by Bonferroni's test: **, $p < 0.01$

Appendix Figure S5. Prom1 is important for quiescent cell re-enter cell cycle

A. Forward and side scatter gating strategy used in the current study.

B. Flow cytometry analysis and quantification of Ki67-FUCCI CLESCs at different cell cycle stages and analysis. As a result, >95% of the events have been included in the analysis. The accuracy of the strategy has been validated by two independent researchers for IF quantification using Fiji 1.0 software.

Appendix Figure S6. Prom1 regulates ciliary dynamics by directly interacting with Hdac6 and Arl13b

A. Parental cells (MDCK) or those stably transfected with Prom1 or K138Q mutant were cultured for 6 days post-confluence (dpc) and then IF stained for AcTub as a marker of primary cilia. Box-whisker plots show the mean fluorescence intensity (MFI) of AcTub associated with primary cilium in the respective cell lines as indicated. More than 300 cilia per experiment were analyzed (n = 3). Mann-Whitney test: **, $p < 0.01$. Note the reduction of AcTub staining in K138Q mutant by comparison to parental cells or those expressing non-mutated Prom1.

B, C. MDCK cell lines as indicated were cultured for 6 (B) or 14 (C) dpc and then IF stained for AcTub as a marker of primary cilia prior the analysis of primary cilium length. Histogram of ciliary length distribution (black, left-y axis) and the quantitation of Prom1$^+$ cells with no cilium (nc, red, right y-axis) are shown. Cilia were classified into three length categories; <3, 3-5 and >5 μm, and numbers of analyzed cilia (black) or cells (red) from 7 (Prom1) or 3 (MDCK, K138Q) independent experiments are indicated in bracket. Mann-Whitney test: **, $p < 0.01$. Data are represented as mean and standard deviation.

D, E. Parental cells (MDCK) or those stably transfected with Prom1 or K138Q mutant were cultured for 6 dpc prior to IF double staining for AcTub and Hdac6. Box-whisker plots show Hdac6/AcTub immunoreactivity ratio (D) associated with cilia in the

corresponding cell lines. At least ten cilia per experiment were analyzed (n = 3). Mann-Whitney test: *, $p < 0.05$. Representative IF micrographs are shown (E).

F, G. K138Q mutant-transfected MDCK cells were incubated for 16 h with fresh media containing tubacin (1 μM), niltubacin (1 μM) or DMSO (1:1000) as vehicle control prior to IF staining for AcTub. Ciliary length under three different experimental conditions was analyzed as fold changes relative to untreated control (F, red line) with the cilium numbers analyzed for each condition indicated under the X-axis. The means and standard deviations are displayed. Maximum projection of 25-35 optical x-y sections (0.38 μm-slices) of the apical part of K138Q mutant-transfected cells labeled with anti-AcTub are shown under different culture conditions as indicated (G). Number of cilia analyzed in indicated in bracket.

H. Expression and subcellular localization of Arl13b upon silencing Prom1. Representative Arl13b immunoblotting of protein fractions derived from CLESCs upon silencing Prom1 using siRNA. Total, cytoplasm and nuclear fractions were probed for Arl13b, as well as Lamin B and GapDH as nuclear and cytoplasm markers, respectively. Molecular mass markers are indicated.

I. Quantification of total Arl13b immunoreactivity associated with siControl vs. siProm1 CLESCs (left panel) and its distribution between cytoplasmic and nuclear fractions. Signals were normalized to the internal control, i.e. Lamin B or GapDH.

J. Representative IF double staining of Arl13b (red) and AcTub (green) as a marker of primary cilia in stem cell region of lower incisor CLE at P7 of WT vs. *Prom1* KO mice. Note the reduction of Arl13b immune signals in Prom1-deficient mice.

Appendix Figure S7. *Prom1* KO incisors have amelogenesis (enamel development) defects

A. Representative stereo view of the lower incisors of 2-month-old WT and *Prom1* KO mice. Arrowheads indicate enamel defects in Prom1-deficient animal.

B, C. SEM analysis of samples indicated in (A). Arrowheads indicated Tomes processes of ameloblasts (C). Note the reduced matrix deposition in the *Prom1* KO animal.

D. Hematoxylin-Eosin staining of ameloblast region of WT vs. *Prom1* KO mice. Note the presence of large vacuoles (arrows) in the cytoplasm of cells from of *Prom1* KO mouse.

E. Representative IF staining of Ameloblastin (green) and Amelogenin (red) on fully differentiated ameloblasts of WT vs. *Prom1* KO mice.

Appendix Figure S8. Prom1 meditates SHH activation through Glis2.

A, B. Representative IF staining of Gli1 (red, A) and Glis2 (red, B) on the stem cell and transit amplifying cell regions in CLE. Samples are counterstained with DAPI (blue). Dotted lines: basement membrane. SCs, stem cells; TACs, transit amplifying cells; Ams, Ameloblasts.

C, D. Real-time RT-PCR profiling of SHH (C) and Gli1-3 (D) mRNA expression in the transit amplifying cell regions of WT vs. *Prom1* KO mice. The results are from triplicated samples in arbitrary values after normalization for *GapDH*. Statistics was performed using one-way ANOVA: **, $p < 0.01$. Data are represented as mean and standard deviation.

E. Real-time RT-PCR analysis of Gli1-3 and Glis1-2 in the human Prom1 and K138Q transfected and MACS sorted CLESCs. Statistics was performed on triplicated samples using one-way ANOVA: **, $p < 0.01$. Data are represented as mean and standard deviation.

F. Representative microCT analysis of enamel (yellow arrows) development of 4 months old female mice with indicated genotypes. Note the reduced length and thickness of the enamel in *Prom1* KO and *Glis2* KO mice.

Appendix Figure S9. Stat3 is downstream target of Glis2

A, B. Real-time RT-PCR analysis of the indicated genes in CLESCs after siProm1 (A, with scrambled siRNA as control) and shGlis2 (B, with empty vector (Plko1) as control) mediated knockdown of gene of interest. The results are in arbitrary values after normalization for *GapDH*. Statistics was performed on triplicated samples using one-way ANOVA: *, $p < 0.05$; **, $p < 0.01$. Data are represented as mean and standard deviation.

C. Representative IF staining of Stat3 (red) on the stem cell and transit amplifying cell regions in CLE. Sample is counterstained with DAPI (blue). Dotted lines: basement membrane. Yellow arrowheads mark approximate stem cell boundaries.

D. The relative positions of Glis2 binding sites on murine *Stat3* promoter with respect to exon1.

E. Quantitative analysis of apoptotic body numbers on the entire CLE region at P7 of WT vs. *Prom1* KO mice using TUNEL assays ($n = 3$). Statistics was performed using one-way ANOVA: **, $p < 0.01$. Data are represented as mean and standard deviation.

F. Apoptotic body quantification on siProm1-treated vs. siControl CLESCs using TUNEL assays. Statistics was performed on triplicated wells using one-way ANOVA: **, $p < 0.01$. Note the increase in the apoptotic bodies upon knockdown (E) or silencing (F) Prom1. Data are represented as mean and standard deviation.

G. Quantitative analysis of Active Caspase3 positive cells in cultured CLESCs in the presence of absence of SHH (100 ng/ml), with or without Prom1 knockdown.

Statistics was performed on triplicated wells using one-way ANOVA: **, $p < 0.01$. Data are represented as mean and standard deviation.

H, I. Representative IF staining of Prom1 (red, H) and AcTub (red, I) in the cervical loop (CL) of WT and *Bmi1* KO mice. Samples are counterstained with DAPI (blue). Dotted lines: basement membrane. In panel I, the stem cell region is shown.

J. Quantification of primary cilium volume in WT vs. *Bmi1* KO mice. 3 animals were used for each both conditions. The number of quantified cilia is indicated (n). Statistics was performed using one-way ANOVA: **, $p < 0.01$.

K. Representative IF staining of Glis2 (red) in the CLE at P7 of WT and *Bmi1* KO mice. Samples are counterstained with DAPI (blue). Dotted lines: basement membrane. Yellow arrowheads mark approximate stem cell boundaries. SCs, stem cells; TACs, transit amplifying cells.

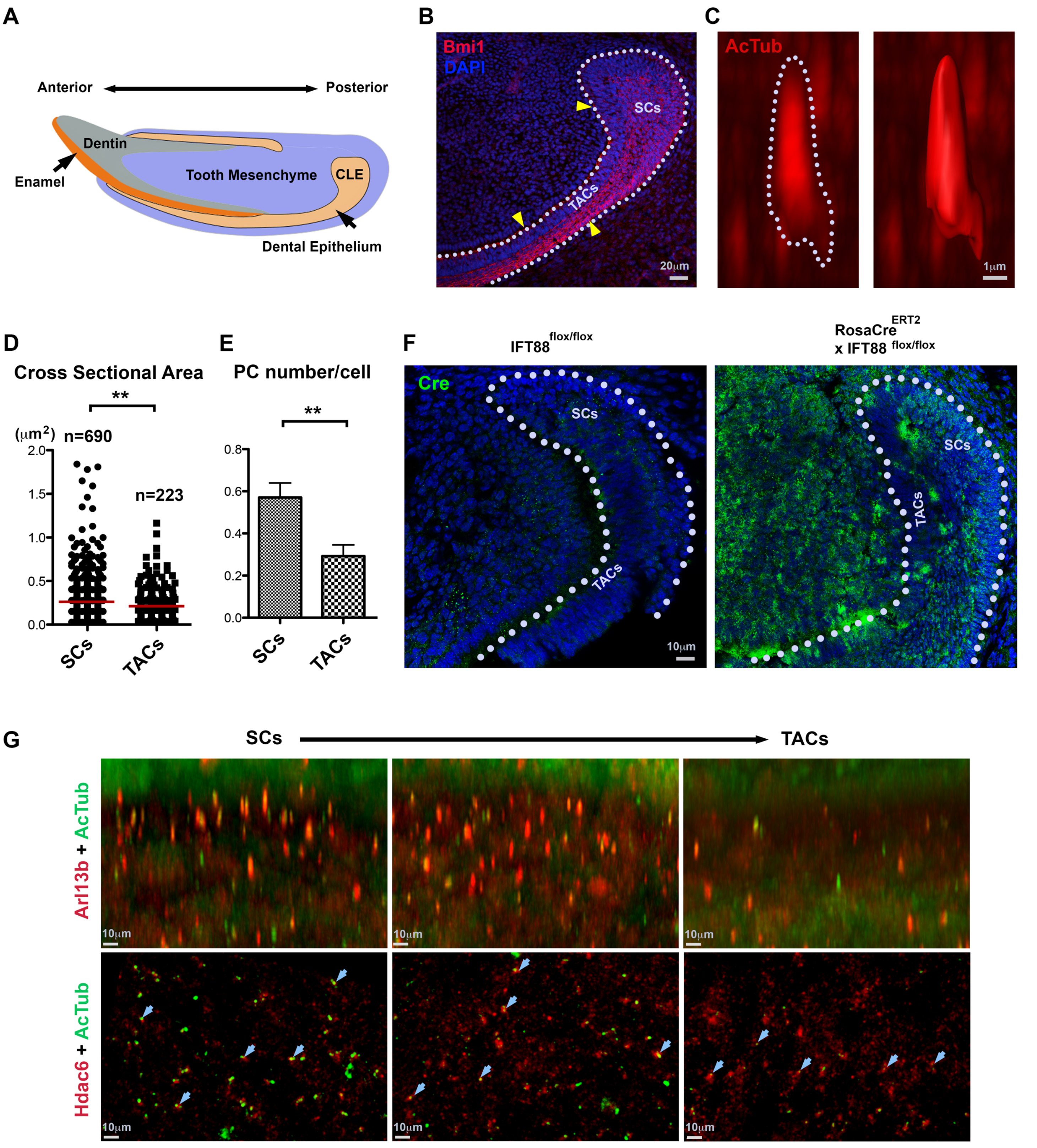

Appendix Figure S1

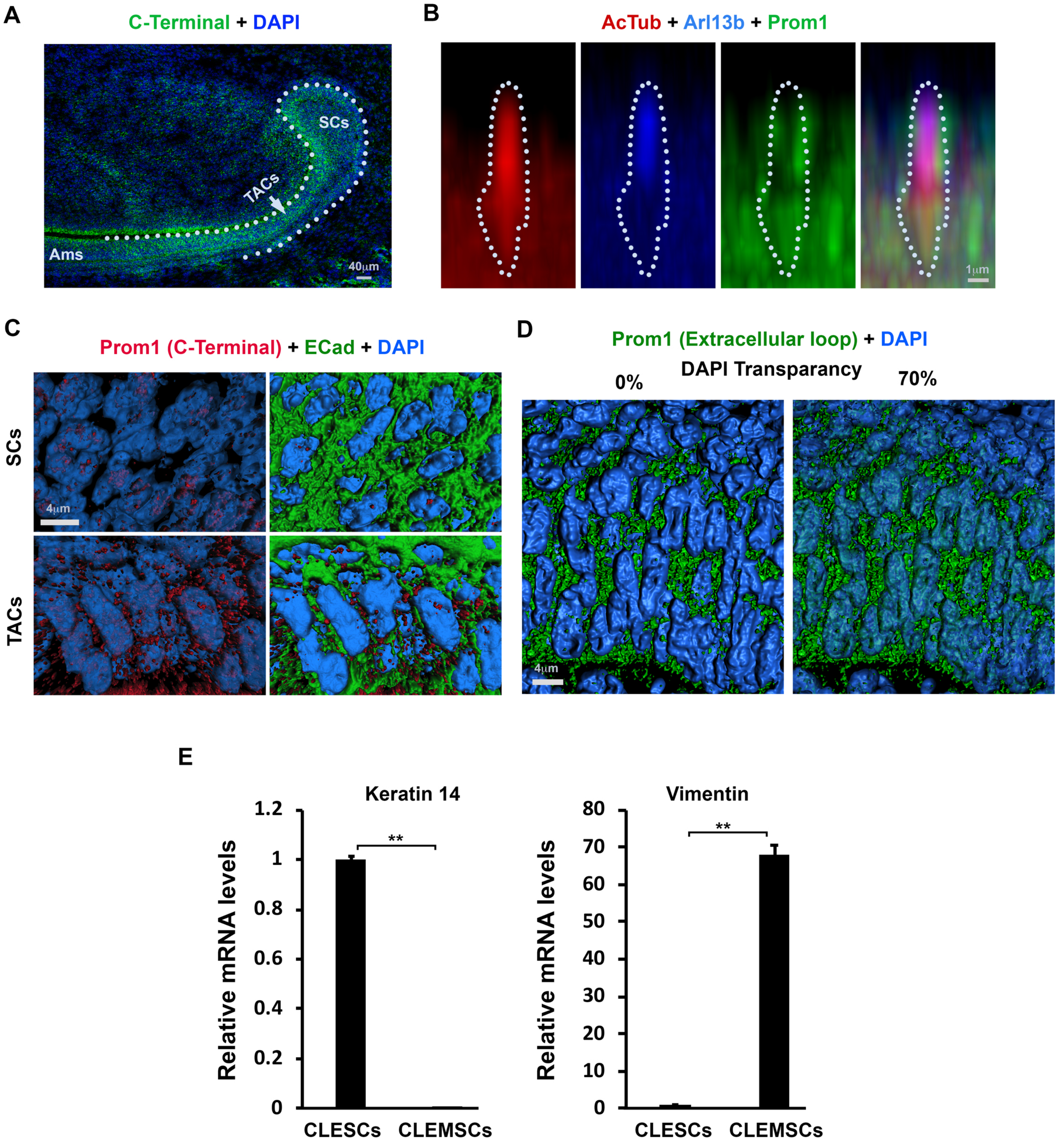

Appendix Figure S2

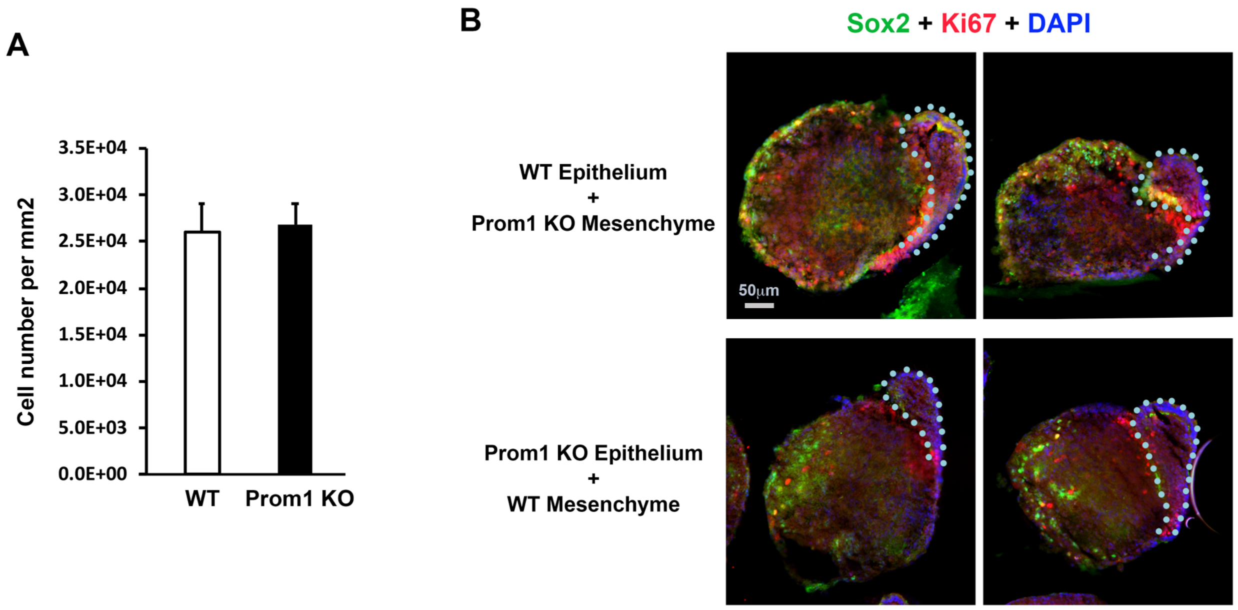

Appendix Figure S3

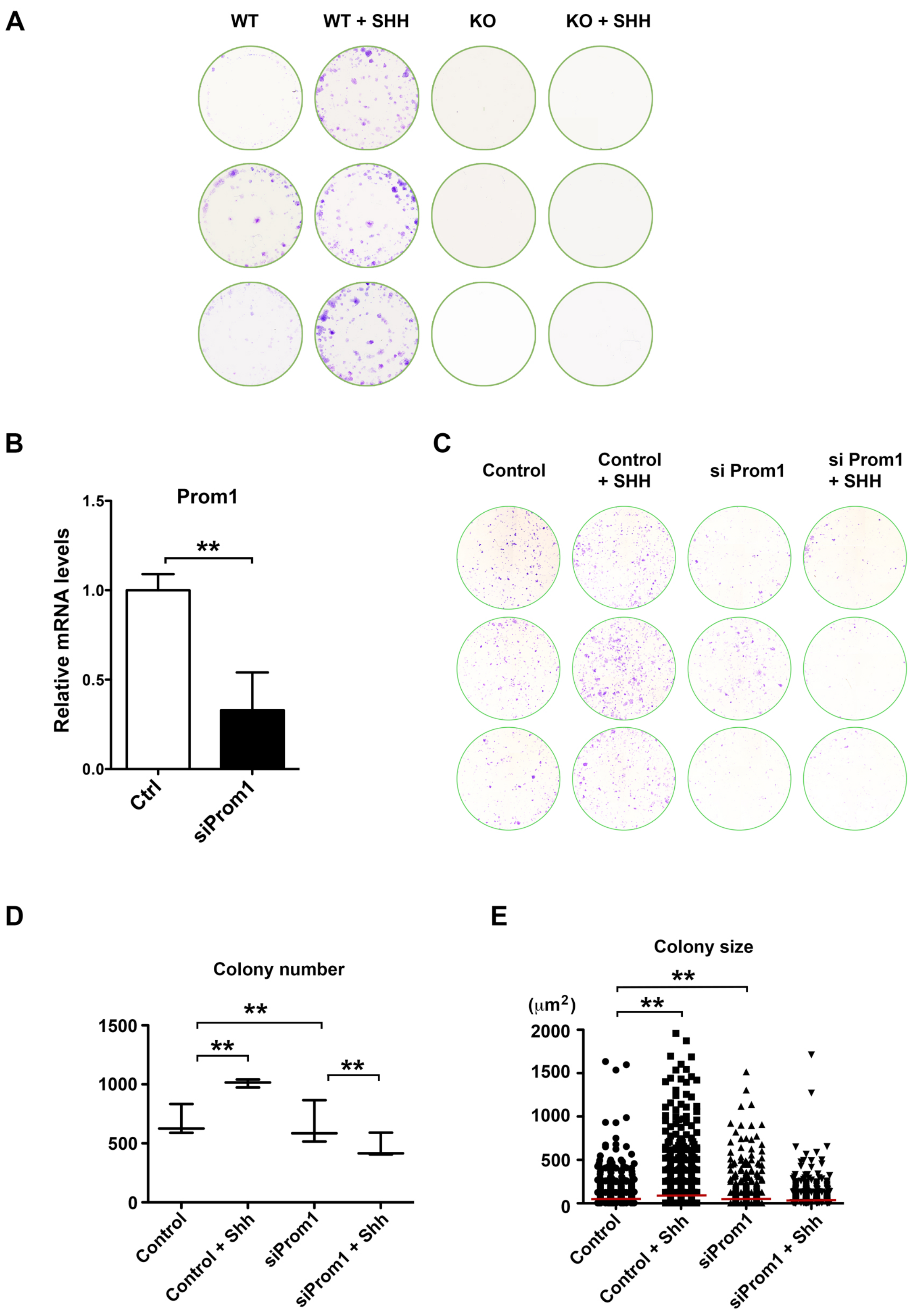

Appendix Figure S4

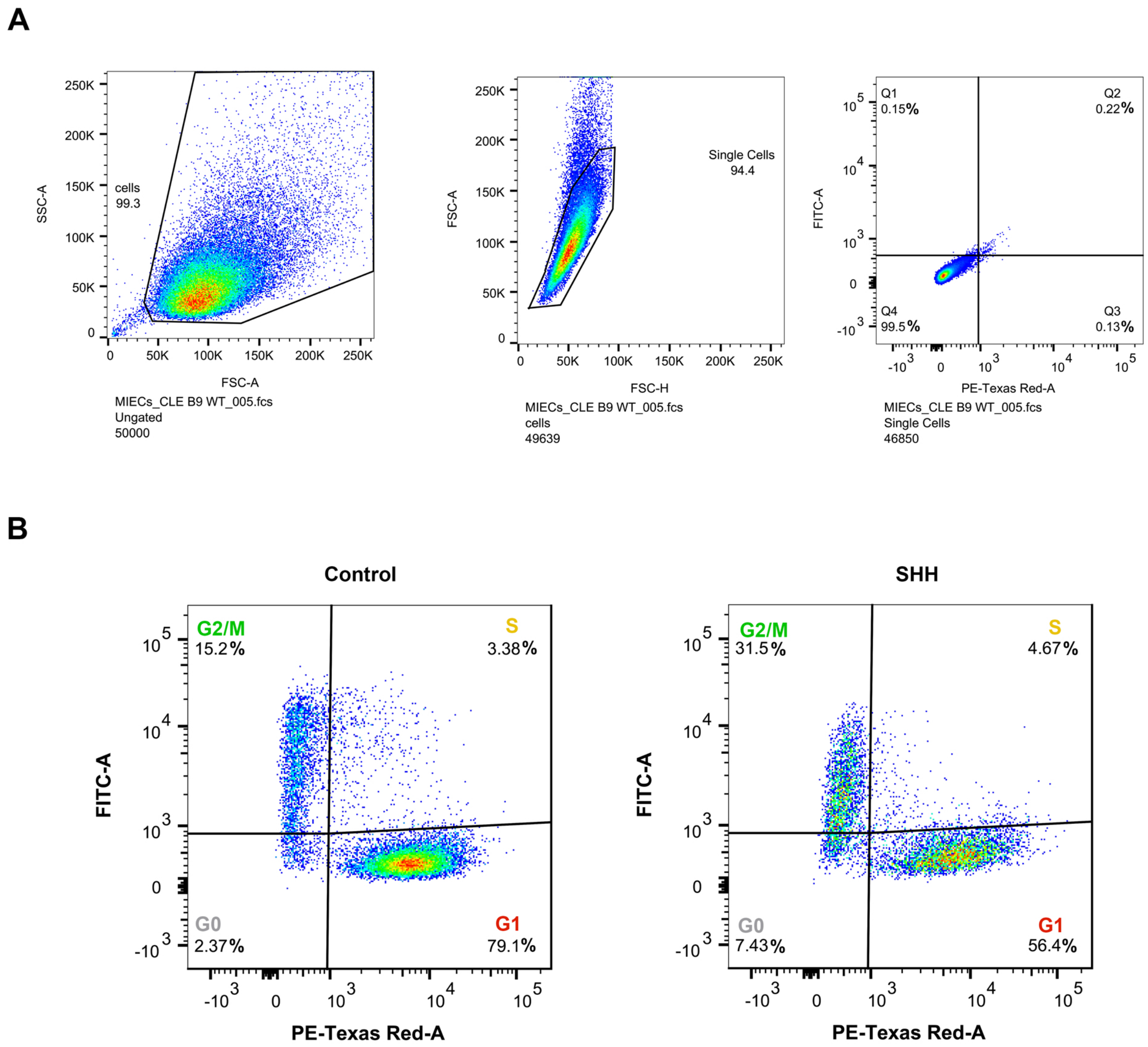

Appendix Figure S5

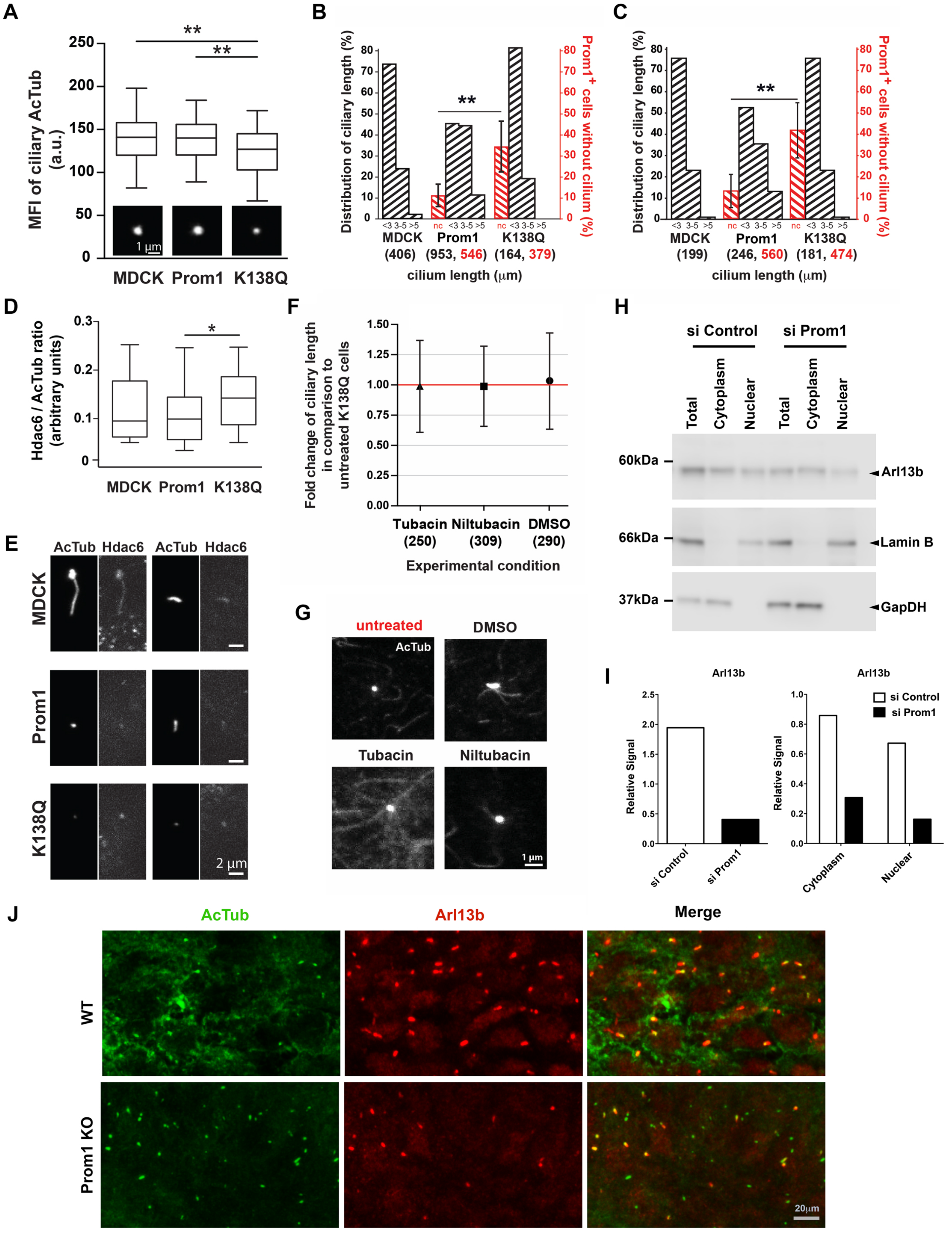

Appendix Figure S6

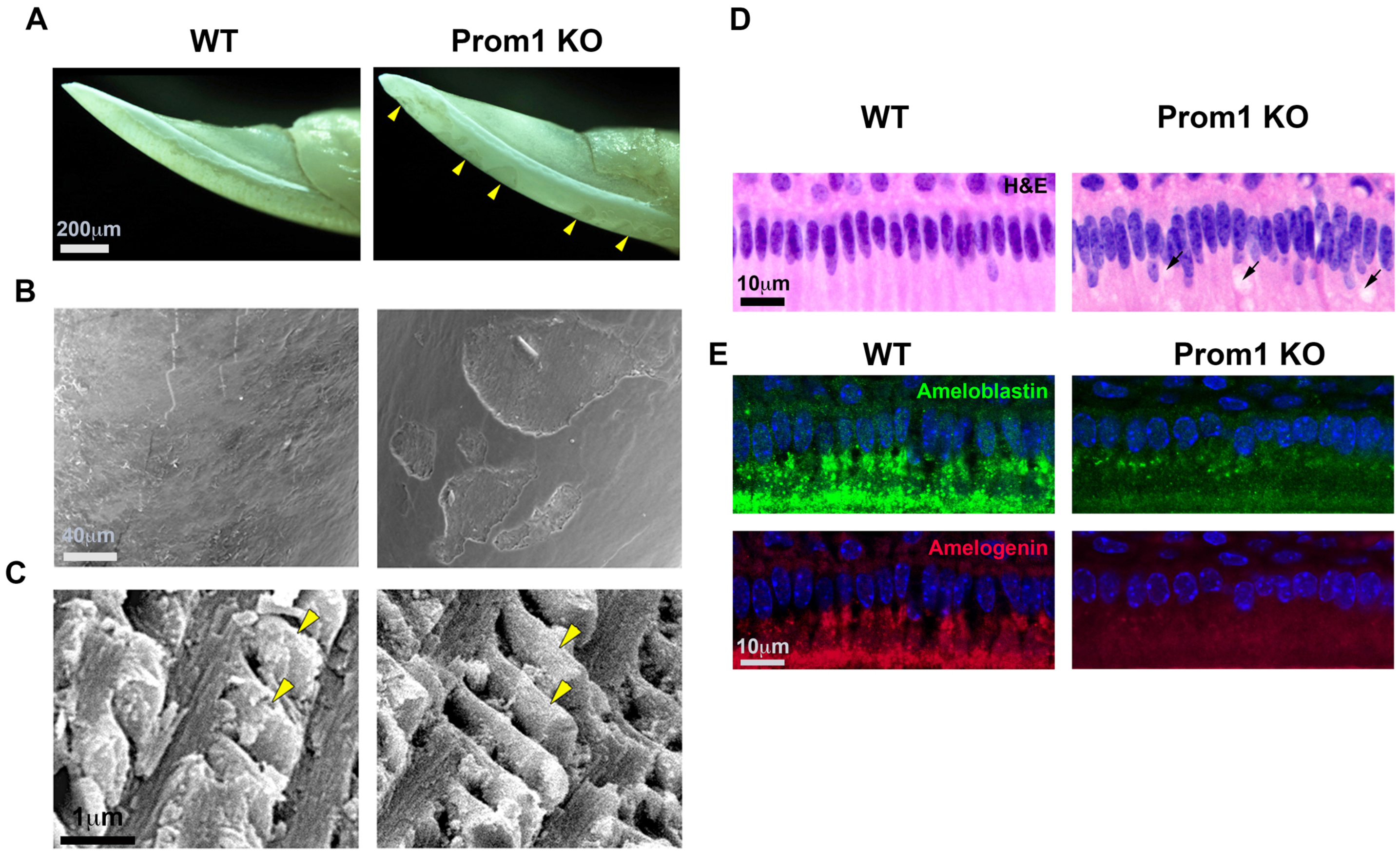

Appendix Figure S7

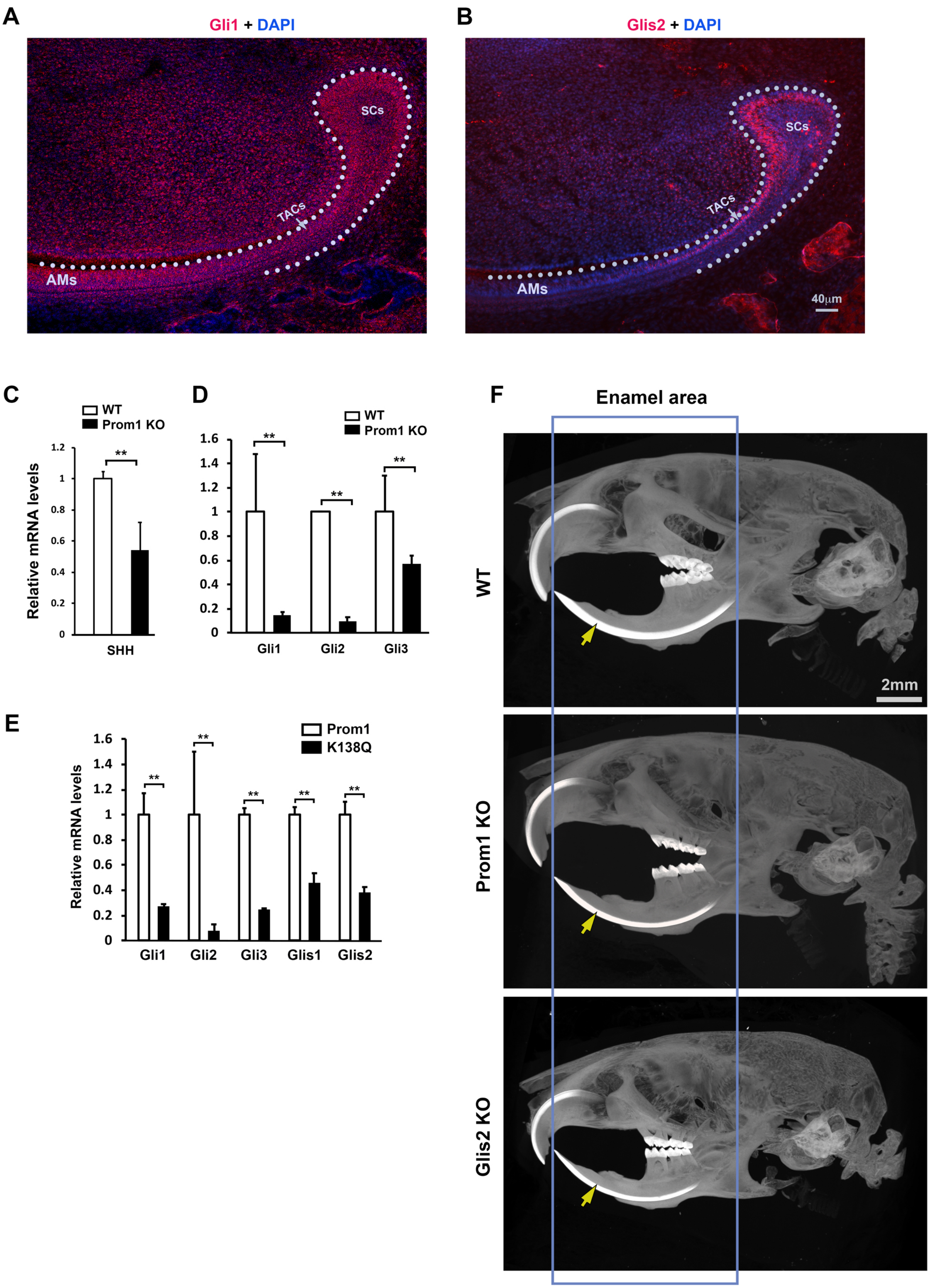

Appendix Figure S8

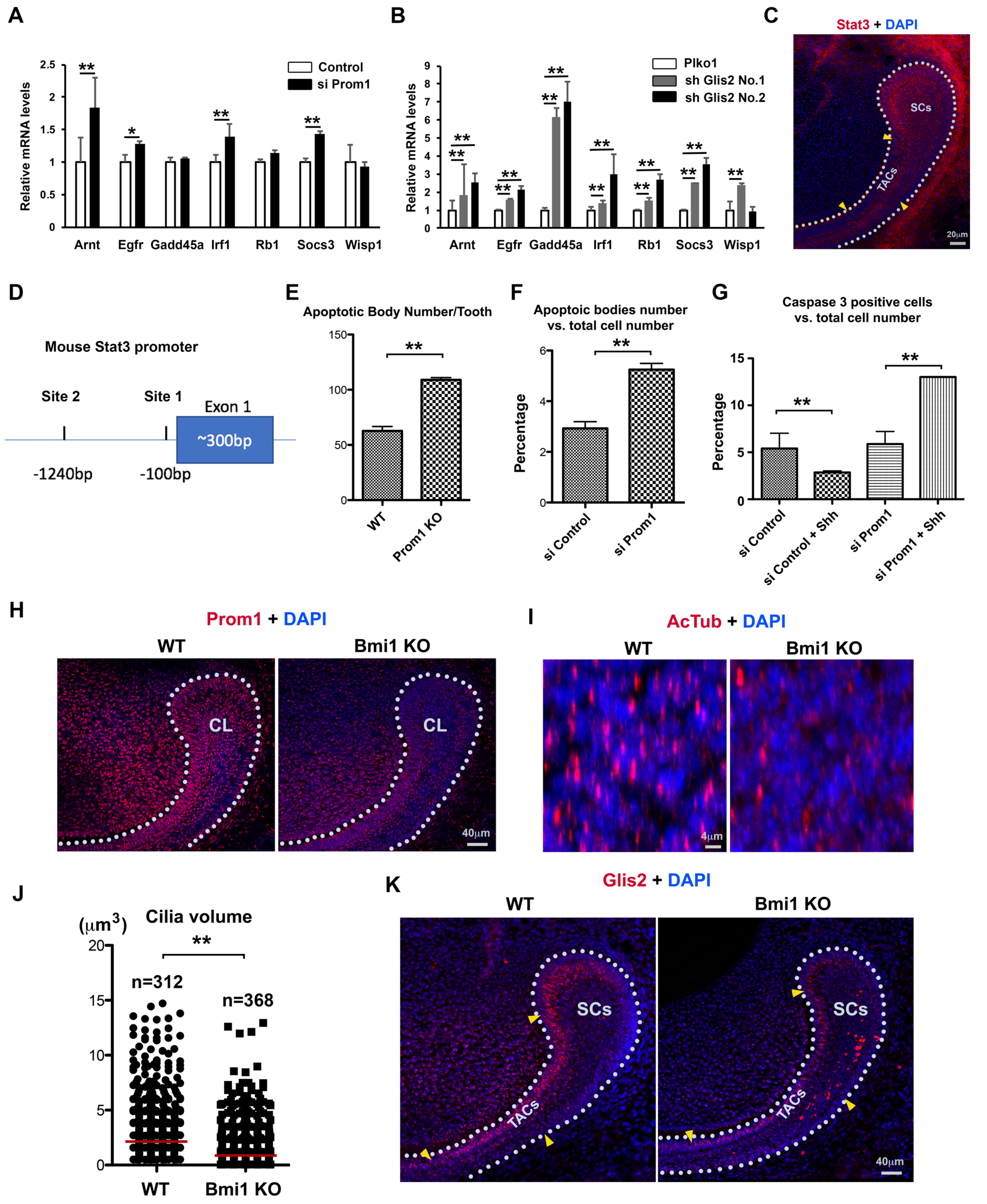

Appendix Figure S9

Appendix Table S1

Primary antibodies							
Target	Immunofluorescence (Diluting facto	Western Blotting	Company	Cat. No.	Lot	Representative publications (showing PMID only)	Validation in this study
Acetylated Tubulin	1/5000 (BH*) 1/1000 (DC**)	1/2000	Sigma-Aldrich	T7451		20972424, 21307093	
Acetylated Tubulin	1/200		Cell Signaling	5335S	Lot4 ref03/2015	26931735, 27002170	
Active Caspase 3	1/200		Cell Signaling	9661	43	28443644, 28289704	
Ameloblastin	1/200		Santa Cruz	sc-33100		27146703	
Amelogenin	1/200		Santa Cruz	sc-32892		21514442	
ARL13b	1/250 (BH*) 1:100 (DC**)	1/400 (BH*) 1/1000 (DC**)	Proteintech	17711-1-AP	00043031	23150559, 27153923	
beta-Actin		1/1000	Cell Signaling	8457	Lot1 ref04/2014	28569748, 27951586	
BMI1	1/100		Santa Cruz	SC-10745	E0316	26788137, 15171791	
CD133 (C-terminal)	1/1000	1/500	Biorbyt	Orb129549	I8429		Fig. 2d-f
CD133 (13A4; Extracellular Loop)	1/50		eBioscience	14-1331-82	e04033-1631	9356465, 15908947, 15316084	
CD133 clone: 80B258		1/2000	Dr Denis Corbeil			18645205, 20717901, 18096722	
CD133 clone: W6B2C1		1/200	Miltenyi Biotec	130-092-395		27634913, 24335200	
CD133/1 (AC133)	1/50		Miltenyi Biotec	130-090-422		9389720, 9389721	
Cre Recombinase	1/100		BioLegend	908001			
ECadherin	1/200		Bio-Techne	BAF748	CXW0315091	24317254, 15994295	
GapDH		1/1000	Santa Cruz	SC-32233	K1216	29042957, 28855737	
Gli1	1/200		Novus Biologicals	NBP1-78259	B-1	24506883, 25799059	
Glis2	1/200		Dr Massimo Attanasio	N/A		16326862, 17289029	
HDAC6 (BH*)	1/200		Dr Tso-Pang Yao	N/A		12024216, 17938201	
HDAC6 (DC**)	1/50		Fisher Scientific	PA1-25455			
Ki67	1/200		Abcam	ab15580	GR1349163-4	28492284, 28281529	
LaminA/C	1/200	1/500	Cell Signaling	4777S	lot1 ref02/2014	24525530, 23319590	
LaminB1	1/200	1/500	Abcam	ab16048	GR302354-1	27391408, 26304990	
Sox2	1/200		R&D Biosystems	AF2018		28226240, 28566702	
STAT3	1/500		Cell Signaling	12640	lot4 ref10/2016	28301550, 26194464	

Details of Prom1 antibody application in the paper	Relevant figure and panel number	Note
CD133 (C-terminal)	2d, 2f-h, 9a, S2a, S2c, S9h	S: Supplemental Data Figure
CD133 (Extracellular Loop)	2a-d, S2b, S2d	
CD133 clone: 80B258	6k-l	
CD133 clone: W6B2C1	7f	
CD133/1 (AC133)	6i-j	

Note: * and ** indicated different conditions used in Corbeil and Hu labs
* BH: Bing Hu's Lab
** DC: Denis Corbeil's lab

Secondary antibodies					
Target	Dilution (for immunolabelling)	Dilution (for Western Blotting)	Company	Cat. No.	Lot
anti-mouse HRP		1/2000	Cell Signaling	7076P2	
anti-rabbit HRP		1/2000	Cell Signaling	7074P3	
anti-rabbit Alexa Fluor 405	1/250		Life Technologies	A31556	1661251
anti-rabbit Alexa Fluor 568	1/500		Life Technologies	A10042	1826664
anti-rat Alexa Fluor 488	1/300		Life Technologies	A21208	1789917
anti-mouse Alexa Fluor 488	1/300		Life Technologies	A21202	
anti-goat Alexa Fluor 488	1/300		Life Technologies	A11055	1182671
anti-mouse Alexa Fluor 488 IgG1	1/500		Life Technologies	A21121	
anti-mouse Alexa Fluor 546 IgG1	1/500		Life Technologies	A21123	
anti mouse Alexa Fluor 488 IgG2b	1/600		Life Technologies	A21141	
anti mouse Alexa Fluor 546 IgG2b	1/600		Life Technologies	A21143	
anti mouse Alexa Fluor 647 IgG2b	1/600		Life Technologies	A21242	
anti rabbit Alexa Fluor 546	1/500		Life Technologies	A21085	

Primers			
Gene name	Forward primer ('5-'3)	Reverse primer ('5-'3)	Product Size
Acsl4	AGAAGGGAAGCAAGGGTGAT	GATTACAAAGAGGGGCGTCA	198
Arl13b	AGAAACGTGAGAGGGCTGAA	TTCTCCACGGTCTGCTTCTT	199
Arnt	CCATCGTTGTGTGGCTACTG	AAGGAGCTCGTTCTCATCCA	201
Axin2	TAATGCTAGGCGGAATGAAG	AACCCATTACAAGCAAACCA	192
Bmi1	CCGCTCTTTCCGGGATCTTT	TGTTCAGGAGTGGTCTGGTT	237
Cdk5r1	TAACAGGATTCCAGCTTTGC	CTAGCGGTCGTTCCTTTACA	201
Cdkn1c	TCTAGGGGAATGGTTGTTGA	GATTTTTGTTGGGCCTCTTT	189
Cdkn2b	CATTTTCTGCAGCTGGATCT	TTCACAGGGGAAGGTACTGA	228
cMyc	CCAGATCCCTGAATTGGAAA	TCGTCTGCTTGAATGGACAG	93
Egfr	GTGATCCAAGCTGTCCCAAT	CTTGGAACTTTTGGCAGACC	198
Gadd45a	TGCGAGAACGACATCAACAT	TCCCGGCAAAAACAAATAAG	200
Gclc	AACACAGACCCAACCCAGAG	CCGCATCTTCTGGAAATGTT	201
Gli1	ATAGGGTCTCGGGGTCTCAA	CGGCTGACTGTGTAAGCAGA	137
Gli2	AGGCAGTCAGTGCCTGGGTAT	ACCGCAGCCAGGGATGA	246
Gli3	TGCCGCTGGCTTGATTGT	TGGCCCGATCTGAAGCAT	241
Glis1	CTCCAAGCATCCACACTGTT	GACAGGATGCCTGAAGCAAG	128
Glis2	CAACGACCATCATGTCAAGC	CGGTTGTGGATCTTCAGGTT	198
Hdac6	TCCCCATTTGATGGCAGTAT	CAGGTCAACACAGCTGAGGA	199
Irf1	CCTGGGTCAGGACTTGGATA	TTCGGCTATCTTCCCTTCCT	202
Jak1	CATGACTCGCTGCATGAACT	TCAACCTTCCCAAAGTGACC	201
Jak2	GTCCACCCGTGGAATTTATG	GAAGGGAAAGGTCCCTGAAG	198
Jak3	GTGGCATCCTGCCTGTTTAT	GGATGGCACTGGTCAAATCT	204
Krt14	CCTGCTGGATGTGAAGACAA	ATCGTGCACATCCATGACCT	190
Lgr5	CCTTCCCTGTGACTGGGTTA	CACTGTTGCCGTCGTCTTTA	201
Lrg1	TCCCATGTCAGTGTGCAGAT	TCAGCCTAGGAGCCGTTTTA	203
p16	CAAGAGCGGGGACATCAAGACATC	AGCTCTGCTCTTGGGATTGGCC	175
p21	CTTGTCGCTGTCTTGCACTC	TCTCTTGCAGAAGACCAATCTG	142
p27	TTGGGTCTCAGGCAAACTCT	TCTGTTGGCCCTTTTGTTTT	152
Prom1	TGTGGATGTCATCAAAGACG	GGGCAGCTCCTGGTTTAAGT	149
Rb1	GCCTCCTACCTTGTCACCAA	TGTCCCAAATGATTCACCAA	199
Shh	TCCACTGTTCTGTGAAAGCA	GTCCAGGAAGGTGAGGAAGT	188
Slc2A1	AAACATGGAACCACCGCTAC	GAGAAGCCCATAAGCACAGC	202
Socs3	GCCCCTTTGTAGACTTCACG	AACTTGCTGTGGGTGACCAT	232
Sox2	AACCCCAAGATGCACAACTC	CTCCGGGAAGCGTGTACTTA	202
Stat3	TGATCGTGACTGAGGAGCTG	TGGCGGCTTAGTGAAGAAGT	203
Stat3 ChIP neg ctrl 1	GCAATTCGACAACAAAAGAAA	TTGAGAAGGGTGTTGTTTCC	250
Stat3 ChIP neg ctrl 2	GACACATGGTTCACATTCTCC	AGTCACTTGATGGCACCTGT	164
Stat3_s1	CACTGCAGTACGCAGAGTTCA	TCCACACTTTCCCACTACTGC	218
Stat3_s2	CAGCAGGACATTCCGCTAAT	GCGAGCGCTGTACTTCCTAA	209
Stat5a	GTGAAGCGCTCAACATGAAA	GAACCACTGCCAGAAGGTGT	200
Stat5b	CCCAAGTCATGGGGAGAGTA	AGCATGCAGATAGGGGACAC	199
Stat6	CCCTGTTCAAGAACCTGCTC	AGCTTTGGCGTTGTTGTCTT	200
Tyk2	ACATGGTCCCTTGGATGTGT	TAGCTTGATGAAGGGGTTGG	199
Vimentin	CCAACCTTTTCTTCCCTGAA	GGTCATCGTGATGCTGAGAA	148
Wisp1	AGATATGTGCCCAGCAGCTT	TTGTACCTGCAGTTGGGTTG	199

esiRNA	
esiRNA (Sigma)	Sequence
esiCD133 EMU055321 SIGMA	ATCGCAGGATGGATTCAGAGGATG<br>TATACGACGATGTTGAGACTGTGC<br>CCATGAAAAATTTGGAAATCGATAG<br>TAATGGTTATCATAAAGATCATTTAT<br>ATGGTGTTCACAATCCTGTTATGAC<br>AAGCCCGTCTCGATACTGACAACT<br>GGAGTTGAAGCTGCTTGAACAACA<br>AGATAGTCAACATGGAAAGCATCAC<br>AGATTTTGGATAGTTTCTGAGTCTT<br>CTAGAACGTTCCAAGTGCAGAAGA<br>AACCTGGTGGAGACTCAGGCGGG<br>CACTAGGAACATGGCATCAGTGGT<br>CTTAGGGATGCACTTTGTCAGGAA<br>TGAACAGTCATCATGGTTATAAGCC<br>ACATATCCATTGCAACTCATGAATG<br>ATTCTCTCCTGTTTTGTTTTTAACT<br>TTTCTTTTTACACTGATTTTCTATTT<br>AGACACTAAAACATATAGGGGTGC<br>TTATTCCCCCTGGATACATTTACCT<br>GTGAACCAGCTATTCCGGT
esiHDAC6 EMU055151 SIGMA	CTGGTACTTCCCATCGCCTATGAG<br>TTTAACCCAGAACTGGTGCTGATC<br>TCAGCTGGCTTTGATGCTGCACAA<br>GGGGATCCGCTGGGGGGCTGCC<br>AAGTAACACCGGAAGGTTATGCCC<br>ACCTCACCCACCTACTGATGGGCC<br>TTGCTGGTGGCCGTATTATTCTTA<br>TTCTAGAGGGTGGATACAATTTGG<br>CATCTATCTCTGAGTCTATGGCTG<br>CCTGCACCCATTCCCTCCTTGGAG<br>ACCCACCACCCCAGCTTACTTTGC<br>TGCGACCGCCACAGTCAGGAGCC<br>CTGGTTTCAATCAGTGAGGTCATC<br>CAAGTCCATCGCAAATACTGGCGC<br>AGTTTGCGGTTGAGTAAAATGGAA<br>GACAAGGAAGAATGCTCTAGTTCT<br>AGGCTTGTCGTCAAGAAGTTGCCC<br>CCAACAGCCAGTCCTGTATCAGCT<br>AAGGAAATGACCACACCGAAAGGA<br>AAGGTTCCTGAAGAAAGCGTGAGG<br>AAGACCA
esiRLUC EHURLUC SIGMA	GATAACTGGTCCGCAGTGGTGGG<br>CCAGATGTAAACAAATGAATGTTCT<br>TGATTCATTTATTAATTATTATGATT<br>CAGAAAAACATGCAGAAAATGCTGT<br>TATTTTTTTACATGGTAACGCGGCC<br>TCTTCTTATTTATGGCGACATGTTG<br>TGCCACATATTGAGCCAGTAGCGC<br>GGTGTATTATACCAGACCTTATTG<br>GTATGGGCAAATCAGGCAAATCTG<br>GTAATGGTTCTTATAGGTTACTTGA<br>TCATTACAAATATCTTACTGCATGG<br>TTTGAACTTCTTAATTTACCAAAGAA<br>GATCATTTTTGTCGGCCATGATTG<br>GGGTGCTTGTTTGGCATTTCATTA<br>TAGCTATGAGCATCAAGATAAGATC<br>AAAGCAATAGTTCACGCTGAAAGT<br>GTAGTAGATGTGATTGAATCATGG<br>GATGAATGG

shRNA			
shRNA (Sigma)	Clone ID	Legacy Clone Name	Target Seq
shCD133-1	TRCN0000115316	NM 008935.1-3396s1c1	CCTGCGGTTTAGAAATAGAAT
shCD133-2	TRCN0000115319	NM 008935.1-1442s1c1	GCTGGAAGAATATGACTCGTA
shCD133-3	TRCN0000115320	NM 008935.1-1800s1c1	CTATTCAATAACCCAGACATT

siRNA (Qiagen)	Cat. No.	Legacy Clone Name
Mm Arl13b 1	SI00937111 (FlexiTube siRNA)	Mm C530009C10Rik 1
Mm Arl13b 2	SI00937118 (FlexiTube siRNA)	Mm C530009C10Rik 2
Mm Arl13b 3	SI00937125 (FlexiTube siRNA)	Mm C530009C10Rik 3
Mm Arl13b 4	SI00937132 (FlexiTube siRNA)	Mm C530009C10Rik 4
